# *Campylobacter jejuni* Actively Invades the Amoeba *Acanthamoeba polyphaga* and Survives within Non Digestive Vacuoles

**DOI:** 10.1371/journal.pone.0078873

**Published:** 2013-11-06

**Authors:** Jenny Olofsson, Diana Axelsson-Olsson, Lars Brudin, Björn Olsen, Patrik Ellström

**Affiliations:** 1 Department of Medical Sciences, Infectious Diseases, Uppsala University, Uppsala, Sweden; 2 Section for Zoonotic Ecology and Epidemiology, School of Natural Sciences, Linnaeus University, Kalmar, Sweden; 3 Marine Microbiology, School of Natural Sciences, Linnaeus University, Kalmar, Sweden; 4 Department of Clinical Physiology, Kalmar County Hospital, Kalmar, Sweden; 5 Department of Medicine and Health Sciences, Linköping University, Linköping, Sweden; 6 Department of Medical Sciences, Clinical Bacteriology, Uppsala University, Uppsala, Sweden; Charité-University Medicine Berlin, Germany

## Abstract

The Gram-negative bacterium *Campylobacter jejuni* is able to enter, survive and multiply within the free living amoeba *Acanthamoeba polyphaga*, but the molecular mechanisms behind these events are still unclear. We have studied the uptake and intracellular trafficking of viable and heat killed bacterial cells of the *C. jejuni* strain 81–176 in *A. polyphaga*. We found that viable bacteria associated with a substantially higher proportion of *Acanthamoeba* trophozoites than heat killed bacteria. Furthermore, the kinetics of internalization, the total number of internalized bacteria as well as the intracellular localization of internalized *C. jejuni* were dramatically influenced by bacterial viability. Viable bacteria were internalized at a high rate already after 1 h of co-incubation and were observed in small vacuoles tightly surrounding the bacteria. In contrast, internalization of heat killed *C. jejuni* was low at early time points and did not peak until 96 h. These cells were gathered in large spacious vacuoles that were part of the degradative pathway as determined by the uptake of fluorescently labeled dextran. The amount of heat killed bacteria internalized by *A. polyphaga* did never reach the maximal amount of internalized viable bacteria. These results suggest that the uptake and intracellular survival of *C. jejuni* in *A. polyphaga* is bacterially induced.

## Introduction

*Campylobacter jejuni* is a major cause of human gastroenteritis worldwide and the most commonly reported zoonosis in the European Union. Infections are associated with contaminated food, primarily consumption of undercooked chicken meat but also unchlorinated water and unpasteurized milk can contain *Campylobacter* spp. [1], [2]. Recent studies have also indicated that water is a more important risk factor for *Campylobacter* infection than previously thought [3]–[5]. A number of studies have enlighten the role of unicellular eukaryotes, particularly free living amoebae for the survival and dissemination of many waterborne bacterial pathogens including *Mycobacterium avium, Legionella pneumophila* and *Vibrio cholerae* [6]–[8]. Free living amoebae are nearly ubiquitous in aquatic environments, were they feed on microorganisms, including bacteria [9]. However, a number of bacteria can resist protozoan grazing and for a few of them, the strategy used to avoid degradation has been described. *L. pneumophila* and *M. avium* survive inside amoebae by their ability to prevent phagosome lysosome fusion whereas *Burkholderia cepacia* survives inside an acidic vacuole that is distinct from the lysosomal compartment [6], [10], [11]. The intra protozoan fate of the bacteria might even differ within a bacterial species i.e. *Escherichia coli*. The virulent serotype *E. coli* O157 is able to invade and multiply within *Acanthamoeba* spp. whereas the avirulent laboratory strain K-12, HB101, is phagocytosed and killed [12], [13]. Both *L. pneumophila* [10], [14] and *M. avium* [6], [15] are known to use the same survival strategy in *Acanthamoeba castellanii* as in human cells.

Uptake studies of *C. jejuni* into human epithelial cells demonstrate that viability is important for bacterial entrance. Konkel et al. showed that inactivation of protein synthesis reduced the amount of *C. jejuni* capable of entering the cells but not of binding to them [16]. The intracellular faith of *C. jejuni* in human epithelial cells was determined in a study by Watson and Galan, where they found that the majority of *C. jejuni* survived by residing in a vacuolar compartment that did not fuse with lysosomes [17]. In contrast, in human macrophages which share features with amoebae, *C. jejuni* could not avoid delivery to lysosomes. Previous studies by us and others have shown that *C. jejuni* are able to invade, survive and multiply within unicellular eukaryotes, mainly of the genus *Acanthamoeba* [18]–[23]. In *A. polyphaga*, the bacteria were able to replicate in co – culture at 37°C under aerobic conditions and survive for more than two months. However, the mechanisms behind these events at a cellular and molecular level are still unclear. In this work we have studied the role of bacterial viability for internalization and intracellular trafficking of *C. jejuni* in the free living amoeba *A. polyphaga*. Our results suggest that *C. jejuni* actively invade the amoeba and escape degradation by avoiding localization to lysosomal vacuoles.

## Materials and Methods

### Bacterial and amoebal cultures

*C. jejuni* reference strain 81–176 was used in all experiments. This wild type strain, kindly provided by Dr. Patricia Guerry, Naval Medical Research Center, USA, was originally isolated from a nine - year - old girl [24]. Before each experiment, bacteria were grown on conventional blood agar plates (Columbia agar II containing 8% [vol/vol] whole horse blood) at 42°C for 24 h in a micro aerobic environment, using a CampyGen gas generating system (CN0025A; Oxoid, Ltd., Basingstoke, United Kingdom) and a BBL GasPak system (BD, Franklin Lakes, NJ). *A. polyphaga* (strain Linc Ap-1) was used in all experiments. The amoeba strain was kindly provided by Bernard La Scola, Université de la Méditerraneé, Marseille, France. Stock cultures of *A. polyphaga* were maintained in peptone yeast glucose (PYG) medium at 27°C in 75 cm^2^ culture flasks (Sarstedt, Nürnbrecht, Germany). For the experiments, *A. polyphaga* were seeded into 24-well culture plates (Fischer Scientific GTF AB, Switzerland) in PYG medium (1 ml/well) and incubated at 27°C for 20 h, until the trophozoites formed confluent layers at the bottom of the wells. Before each experiment, the medium in all wells was gently removed, with care taken not to disturb the attached amoebae in the bottom of the wells, and replaced with 1 ml fresh PYG medium.

The amoebal concentration, viability and the amount of trophozoites was monitored during all time points observed in the experiments. The amoebal concentration was approximately 10^6^/well and the viability varied between 70–80% during the whole experimental period (TC10™ Automated Cell Counter, Bio-Rad Laboratories AB, Sweden). In microscope observations the confluency of trophozoites was estimated to 100% at the start of the experiments and decreased with time to approximately 95% (24 h), 90% (48 h), 70% (72 h) and 60% (96 h).

### Heat inactivation of bacteria

Bacteria were harvested and suspended in 1 ml of PYG medium (Live/Dead staining) or PBS (cyanoditolyl tetrazolium chloride (CTC) staining; Polyscience, Eppelheim, Germany) to a final concentration of 10^8^ CFU/ml, and the solution was divided in two. One part was heat inactivated in a water bath (Heto DT Hetotherm, Denmark) for 45 min at 70°C; the other part was kept at 4°C. To exclude viability among heat inactivated cells, 100 µl of the sample was plated on blood agar plates and checked for bacterial growth. For Live/Dead stained bacteria heat inactivation took place before staining, whereas for CTC stained bacteria heat inactivation was done after staining

### Staining of the bacteria

Bacteria were stained using two different approaches depending on the experiment. For the quantification experiment bacterial suspensions, both heat killed and viable, were stained using Live/Dead (Bacterial Viability Kit; Molecular probes) according to the manufacturers instructions with incubation for 15 min at room temperature in the dark. Bacteria with intact membranes fluoresced green (live), whereas bacteria with damaged membranes fluoresced red (dead). For localization experiments, bacterial suspensions were stained using CTC solution according to the manufacturer's instructions with incubation for 1 h at room temperature in the dark. CTC was used instead of the Live/Dead stain because it has red fluorescence which gives a better contrast to the green Alexafluor-488-dextran containing lysosomes. After incubation, CTC and Live/Dead stained bacterial cells were centrifuged at 8,000×g for 10 min. The pellet was resuspended in PYG (Live/Dead stained bacteria) or PBS (CTC stained bacteria), and used as stained stock solution.

### Quantification of adhered/internalized viable and heat killed *C. jejuni* in *A. polyphaga*

To each well, 50 µl of Live/Dead stained bacterial suspensions, heat killed and viable, were added (at a multiplicity of infection of 20 bacteria per amoeba). The co-culture plates were then incubated in the dark at room temperature under aerobic conditions. Samples were taken at 1 h, 24 h, 48 h, 72 h and 96 h and the number of viable and heat killed *C. jejuni* adhered/internalized to each amoeba were counted. The samples representing the different time intervals were taken from separate wells; one well was dedicated for each time interval. 1 ml of co-culture was removed from the bottom of the wells and the sample was washed and centrifuged twice at 400×g for 8 min. Thereafter, samples were resuspended in PYG medium and analyzed manually in a fluorescence microscope (Axioskop microscope; Zeiss, Germany). Before sampling, wells were washed three times with PYG medium to exclude extracellular non attached bacteria and excessive stain. From each sample 50–100 amoebae trophozoites were analyzed.

### Intracellular localization of viable and heat killed *C. jejuni* in *A. polyphaga*

Stained (CTC) viable and heat killed bacteria were added to separate wells, with the amount of 50 or 100 µl/well (at a multiplicity of infection of 20 and 100 bacteria per amoeba respectively). The co-culture plate was centrifuged at 200×g for 5 min (Allegra 25R, Beckman Coulter) to compensate for the delayed association of heat killed bacteria with amoebae [17] and then incubated in the dark at room temperature under aerobic conditions. After 1 h Alexafluor-488 labeled dextran (1 mg/ml; Molecular Probes) was added to the wells. Samples were taken at 1 h, 24 h, 48 h and 72 h and the number of viable and heat killed *C. jejuni* outside and inside dextran filled vacuoles were counted in each amoeba. Apart from the 1 h sample, that was washed before the dextran was added, sampling procedure was the same as for the quantification of adhered/internalized bacteria. From each sample 50–100 amoebae trophozoites were analyzed. To further compensate for differences in the trophozoites' uptake of viable and heat killed bacteria and to make sure that ongoing active uptake did not interfere with the monitoring of the intracellular fate of *C. jejuni*, an additional localization experiment was done with only viable bacteria as starting material. The experiment was performed as described above, but after co-incubation with viable *C. jejuni* and amoebae, extracellular bacteria were removed by gently washing the wells three times in PYG. Before dextran was added, wells with bacteria intended for inactivation were incubated in PYG supplemented with 8 mg/L erythromycin (Sigma-Aldrich, Stockholm, Sweden) for 1 h in the dark at room temperature. This antibiotic concentration inhibited bacterial growth and showed no visual negative effects on amoebal trophozoites (data not shown). Wells with bacteria intended to remain viable were incubated in PYG medium without the antibiotic. Samples were taken at 24 h and 48 h and the number of viable and erythromycin treated *C. jejuni* outside and inside dextran filled vacuoles were counted in each amoeba.

### Statistical analysis

Counts were Poisson distributed and logarithmic values were therefore used in the analysis by adding a constant 0.5 to manage zeros. Each amoeba was judged both concerning viable and heat killed bacteria and thus repeated measures ANOVA with Duncańs post test was used for the quantification study with time interval as categorical predictor. When analyzing the fraction of trophozoites that associated with bacteria, each amoeba was categorized as having either 1) only viable bacteria, 2) only heat killed and 3) both viable and heat killed bacteria inside. Differences between viable and heat killed amoeba categories (1 and 2) for every time interval were then analyzed by an exact test corresponding to McNemaŕs paired Chi 2 modification first proposed by Liddell (1983) [25]. In localization studies each amoeba was judged both concerning inside digestive vacuoles and inside non digestive vacuoles and thus repeated measures ANOVA with Duncańs or Tukeýs post test was used, with time interval and viable or heat killed or erythromycin treated bacteria as categorical predictor. Confidence intervals for binary data were calculated using the binomial distribution. Data were analyzed using Statistica version 10 (Statistica; StatSoft®, Tulsa, OK, USA) or GraphPad Prism version 5 and p values<0.05 were considered significant.

## Results

### Bacterial viability influences the internalization of *C. jejuni* into *A. polyphaga*

**Figure 1:**
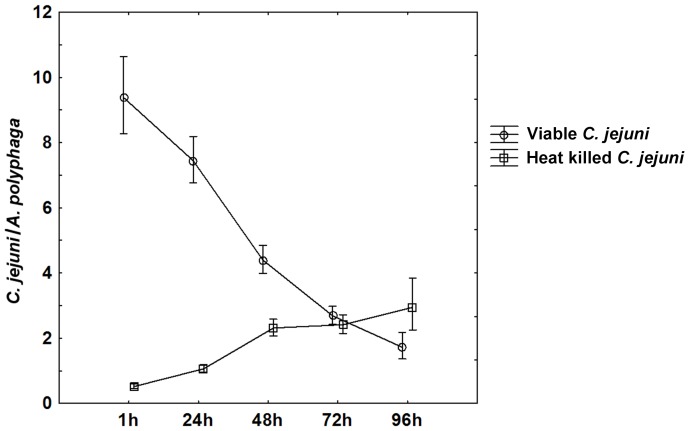
Adhered/internalized viable and heat killed *C. jejuni* per *A. polyphaga* trophozoite. After 1-incubation the majority of amoeba associated viable *C. jejuni* were found on the surface of trophozoites but at 24 h and thereafter, the majority were found in intracellular vacuoles. The number of viable bacteria internalized into *A. polyphaga* trophozoites was significantly higher than for heat killed bacteria during the first 48 h. Data are based on three to five independent experiments for samples taken at 1 h–72 h and on one experiment for 96 h. Mean±95 confidence interval.

Co-incubation of *A. polyphaga* with viable or heat killed *C. jejuni* 81–176 showed that both viable and heat killed bacteria were internalized. However, the kinetics of internalization and the total number of internalized bacteria were dependent on bacterial viability. Figure 1 shows the number of *C. jejuni* associated with *A. polyphaga* trophozoites at different time points. After one hour of co-incubation approximately 90% viable *C. jejuni* were found attached to the amoebal surface and 10% inside clearly defined intracellular vacuoles. At later time points (24 h–96 h) of co-incubation the ratio between adhered and internalized viable bacteria had changed so that more than 80% of the amoeba associated viable *C. jejuni* were observed inside intracellular vacuoles. Heat killed *C. jejuni* on the other hand were hardly seen attached to the amoebal surface at any time point, but was only found inside amoebal vacuoles. The internalization of viable bacteria into *A. polyphaga* was significantly higher compared to heat killed bacteria after 1 h, 24 h and 48 h co-incubation (repeated measures ANOVA with Duncańs post test; (1 h) p<0.001, (24 h) p<0.001, (48 h) p<0.001; Fig. 1). After 1 h the number of viable *C. jejuni* declined from 9.4 bacteria/amoeba to 1.7 bacteria/amoeba at 96 h. In contrast, the internalization of heat killed *C. jejuni* into *A. polyphaga* trophozoites increased with time from 0.5 bacteria/amoeba at 1 h to 2.9 bacteria/amoeba at 96 h. The highest number of bacteria internalized into *A. polyphaga* trophozoites at a certain time point was significantly higher for viable bacteria, (9.4 bacteria/amoeba after 1 h) than for heat killed bacteria (2.9 bacteria/amoeba after 96 h) (repeated measures ANOVA with Duncańs post test, p<0.001; Fig. 1).

### Viable *C. jejuni* were internalized into a higher proportion of *A. polyphaga* trophozoites than heat killed bacteria

**Figure 2:**
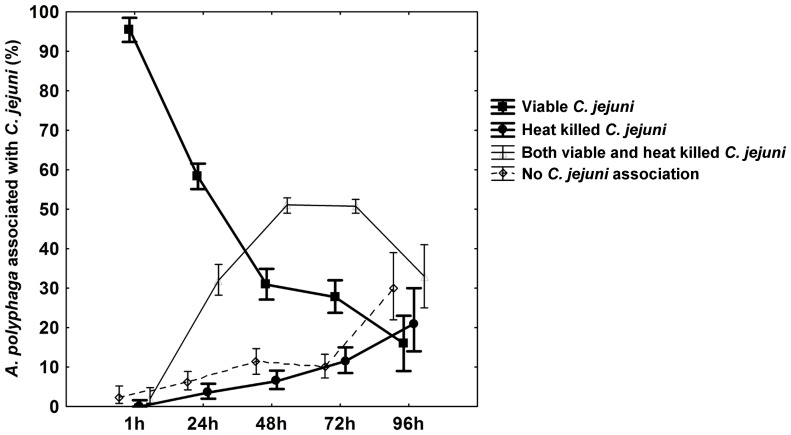
*A. polyphaga* trophozoites associated with *C. jejuni*. The fraction of *A. polyphaga* trophozoites associated with *C. jejuni* are shown as % of all counted trophozoites at 1 h, 24 h, 48 h and 72 h after infection. The trophozoites registered at each time point fall into one of the following categories: Trophozoites associated with viable *C. jejuni* only (squares), associated with heat killed *C. jejuni* only (circles), associated with both viable and heat killed *C. jejuni* (triangles) or associated with no *C. jejuni* (diamonds). Among the trophozoites associated with both viable and heat killed *C. jejuni,* the amount of viable *C. jejuni* were dominating at all time points except at 96 h. Data are based on three to five independent experiments for samples taken at 1 h–72 h and on one experiment for 96 h. Mean±95 confidence interval.

After co-incubation of *A. polyphaga* with viable or heat killed *C. jejuni* we found that the fraction of trophozoites with attached or internalized viable bacteria was significantly higher than the fraction associated with heat killed bacteria. After one hour of co-incubation 96% of the *A. polyphaga* trophozoites were associated with viable *C. jejuni* compared to 0% of the trophozoites associated with heat killed *C. jejuni* (Liddelĺs exact test; (1 h) p<0.001; Fig. 2). The fraction of trophozoites associated with both viable and heat killed *C. jejuni* was initially low but increased with time. Importantly, when the proportion of viable/heat killed *C. jejuni* was analyzed among trophozoites of this fraction the amount of viable bacteria were dominating at all time points except at 96 h (Liddelĺs exact test; (1 h) p<0.001, (24 h) p<0.001, (48 h) p<0.001, (96 h) p = 0.027; Fig. 2), where the number of heat killed *C. jejuni* exceeded the number of viable.

### The intracellular trafficking of internalized *C. jejuni* in *A. polyphaga* is influenced by bacterial viability

**Figure 3:**
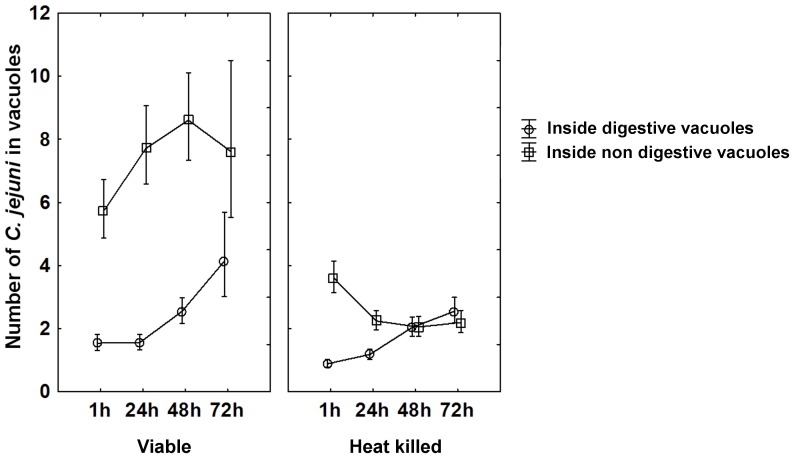
Intracellular localization of viable and heat killed *C. jejuni* in *A. polyphaga*. The number of viable and heat killed *C. jejuni* found inside digestive- (circles) and non digestive (squares) vacuoles are shown after 1 h, 24 h, 48 h and 72 h of co-incubation. Viable *C. jejuni* were found inside non digestive vacuoles to a significantly larger extent than heat killed bacteria. This difference was not observed inside digestive vacuoles. Data for all time points are based on two to three independent experiments, except for viable *C. jejuni* and *A. polyphaga* at 72 h that was based on one experiment. Mean±95 confidence interval.

To determine the role of bacterial viability for the intracellular trafficking of *C. jejuni* after internalization into *A. polyphaga* trophozoites, the intracellular localization was compared between viable and heat killed *C. jejuni* using Alexafluor-488 labeled dextran as a marker for digestive vacuoles. Dextran is internalized and processed via the endocytic pathway ending up in degradative phagolysosomes both in human cells and the widely used amoebozoa species *Dictyostelium discoideum* [26], [27] and vacuoles with green fluorescence are determined as degradative. After 1 h of co-culture viable bacteria were found inside non digestive vacuoles as well as inside digestive vacuoles. However, the number of viable bacteria inside non-digestive vacuoles was significantly higher than inside digestive vacuoles at 1 h (repeated measures ANOVA with Duncańs post test p<0.001; Fig. 3) and increased further with prolonged co-incubation. The number of viable bacteria inside digestive vacuoles on the other hand, increased slightly after 48 h of co-incubation but remained significantly lower than those inside non-digestive vacuoles at all time points. In contrast, heat killed *C. jejuni* were found in low numbers confined to non digestive vacuoles after 1 h of co-incubation with numbers decreasing further with time, whereas the uptake of heat killed bacteria in digestive vacuoles showed kinetics similar to that of viable *C. jejuni*. The fractions of the total number of viable or heat killed bacteria that were localized in non digestive vacuoles after 1 h were 79% and 80% respectively. This fraction remained high for viable bacteria after 48 h of co-incubation and decreased thereafter to 65% at 72 h, whereas for heat killed bacteria it decreased to 65% already after 24 h and declined further to 46% after 72 h. Significant differences between the fractions of viable and heat killed bacteria localized in non digestive vacuoles were observed at time points 24 h (p<0.001), 48 h (p<0.001) and 72 h (p = 0.001). Absolute numbers of bacteria observed inside non digestive vacuoles were significantly higher for viable bacteria than for heat killed bacteria at all time points (repeated measures ANOVA with Duncańs post test, (1 h) p<0.001, (24 h) p<0.001, (48 h) p<0.001, (72 h) p<0.001; Fig. 3). This difference was much less pronounced inside digestive vacuoles, where similar amounts of viable and heat killed bacteria were found at all time points (Fig. 3). To compensate for differences in uptake efficiency between viable and heat killed *C. jejuni* an additional localization experiment was done. After 1 h of co-culture, extracellular, initially viable, bacteria were washed away and intracellular bacteria were inactivated with erythromycin and compared with non inactivated bacteria regarding their intracellular localization. The total amounts of internalized bacteria were somewhat lower in this experiment as internalization was restricted to 1 h by removing extracellular bacteria. However, we still found differences in the intracellular localization between viable and erythromycin inactivated bacteria. After 24 h and 48 h, similar amounts of viable bacteria were found within digestive vacuoles and non digestive vacuoles (repeated measures ANOVA with Tukeýs Multiple Comparison Test, (24 h) p>0.05, (48 h) p>0.05). However, bacteria inactivated with erythromycin were more likely to be found inside digestive vacuoles after 24 h (p<0.05) and 48 h (p<0.05), than in non digestive vacuoles.

### Viable and heat killed *C. jejuni* are taken up into different types of *A. polyphaga* vacuoles

**Figure 4:**
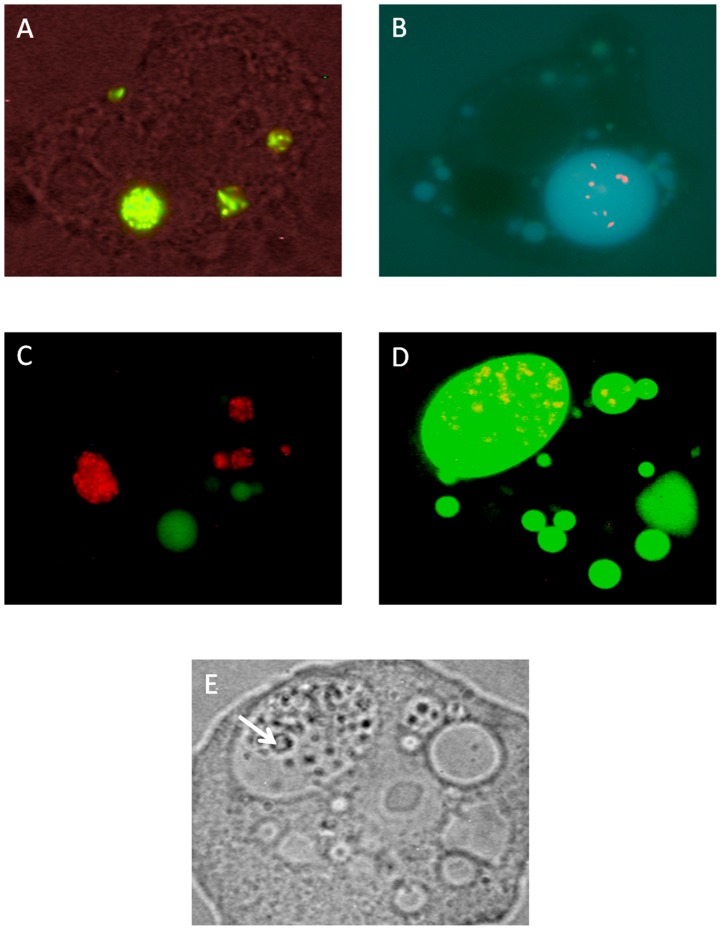
Viable and heat killed *C. jejuni* are taken up into different types of *A. polyphaga* vacuoles. (A) Live/Dead stained viable (green) *C. jejuni*, confined to tight vacuoles. (B) Live/Dead stained heat killed (red) *C. jejuni* residing in giant spacious vacuoles. (C) Vaculoes with CTC stained viable (red) *C. jejuni* do not co-localize with Alexa fluor-488 labeled dextran filled vacuoles (green). (D) Vaculoes with CTC stained heat killed (red) *C. jejuni* have taken up Alexa fluor-488 labeled dextran (green). (E) In contrast to non digestive vacuoles, giant digestive vacuoles contained smaller vesicles (arrow). Picture D and E are from the same amoeba. ZEISS Axioskop (Germany)×63, FI 450–490 (FT 510, LP 520) and a Nikon camera COOLPIX 995 was used for fluorescence images A, C, D and bright field image E. OLYMPUS BX 50 (Japan) ×100 and an OLYMPUS camera DP 50 was used for fluorescence image B.

The morphology of the intracellular vacuoles differed between *A. polyphaga* trophozoites co-incubated with viable *C. jejuni* compared to those co-incubated with heat killed bacteria. Viable bacteria were predominantly localized to small vacuoles where the bacteria were densely packed (Fig. 4A). These vacuoles were distributed throughout the amoeba. Heat killed bacteria, on the other hand, were mostly gathered in one giant, spacious vacuole within the amoeba, often located near the amoebal membrane (Fig. 4B). A common feature for these giant vacuoles was the existence of smaller vesicles within them (Fig. 4E), typical for mature late endosomes and phagolysosomes in the endocytic pathway [28], [29]. To ensure that giant vacuoles belonged to the endocytic pathway, Alexafluor-488 labeled dextran was added to the co-cultures. Fluorescence microscopy revealed that giant vacuoles with lots of heat killed *Campylobacter* were dextran filled (Fig. 4D), whereas viable bacteria were predominantly gathered in vacuoles that did not contain dextran (Fig. 4C).

In co-cultures with viable *C. jejuni* and *A. polyphaga* trophozoites we observed numerous vacuoles containing aggregated viable bacteria in the surrounding medium. These vacuoles showed the same morphology as the vacuoles with viable *C. jejuni* residing inside the amoeba and were most likely expelled from the *A. polyphaga* trophozoites. In co-cultures with heat killed *C. jejuni*, only few vacuoles were observed in the surrounding medium.

## Discussion

Free living amoebae can function as a reservoir host for many species of bacteria including important human pathogens [30], [31]. It has previously been shown by us and others that *Campylobacter* spp. can survive and replicate in amoebae of the genus *Acanthamoeba* [18], [20], [22]. Here we have studied the uptake and intracellular trafficking of the *C. jejuni* strain 81–176 in *A. polyphaga* and found that the kinetics of internalization, the total number of internalized bacteria as well as the intracellular localization of internalized *C. jejuni* were dependent on bacterial viability. Furthermore, the number of *A. polyphaga* trophozoites that associated with bacteria was also strongly influenced by bacterial viability. These results suggest that the uptake and intracellular survival of *C. jejuni* in *A. polyphaga* is bacterially induced.

To determine the importance of bacterial viability for internalization and intracellular trafficking in the amoebal species *A. polyphaga* we compared these processes between viable and heat killed cells of the *C. jejuni* strain 81–176. We found that the kinetics of internalization was quite different between viable and heat killed bacteria in that viable bacteria were taken up at a high rate up to 24 h of co-culture with a decline thereafter, whereas the heat killed bacteria showed a significantly lower initial rate of internalization. This result suggests that *A. polyphaga* can ingest both viable and heat killed *C. jejuni*, but that viable bacteria were taken up more efficiently, indicating a bacterially induced invasion. A similar pattern has been observed for *B. cepacia* where viable, but not heat killed bacteria were internalized into *A. polyphaga* [32]. Konkel et al. found that *C. jejuni* invasion of human epithelial cells required viable bacteria as invasion was completely abolished for heat inactivated *C. jejuni* [16]. In the surrounding media of co-cultures with viable *C. jejuni* we observed numerous vacuoles containing aggregated viable bacteria. These excreted vacuoles likely account for the decline in the number of internalized viable *C. jejuni* observed after prolonged co-culture (Fig 1). Vesicles with viable bacteria released into the extracellular medium have also been reported for *L. pneumophila*, *B. cepacia* and *Parachlamydia acanthamoeba* when co-cultured with *Acanthamoeba* spp. and have been considered as a route of transmission of these bacteria [30], [33]–[35]. The proportion of trophozoites with internalized viable bacteria was dramatically higher during the first 24 h compared to those with internalized heat killed *C. jejuni*. This further indicates that grazing of heat killed bacteria is a slower process than the invasive internalization of viable *C. jejuni*. Grazing appeared to be a specific process, initially restricted to a subset of trophozoites, whereas the invasion process seemed more general and affected virtually the whole amoebal population. Pickup et al. showed that *A. castellanii* grazing of *E. coli* was ineffective when co-incubated with bacteria in suspension, but increased with prolonged incubation time due to formation of bacterial aggregates [36]. This might be a possible explanation for the increased grazing rate of heat killed bacteria with time observed in our experiments.

Studies of intracellular localization using the lysosomal marker dextran showed that the majority of the viable *C. jejuni* were localized in non acidic vacuoles and only a smaller fraction in acidic vacuoles. This difference was not observed for heat killed *C. jejuni* where the numbers of bacteria inside and outside acidic vacuoles were equal. Baré et al. have earlier reported viable *C. jejuni* both within acidified and non acidified vacuoles of *A. castellanii*, but did not provide a quantitative comparison [37]. After prolonged incubation heat killed bacteria gathered in giant, dextran filled vacuoles characteristic of amoebal lysosomes [27], [38], but hardly any viable bacteria could be found in such compartments. These results indicate that both viable and heat killed bacteria were processed for degradation in acidic vacuoles, but that viable bacteria could to a larger extent escape this degradative pathway and the giant lysosomes in particular. By inactivating intracellular *C. jejuni* with the protein synthesis inhibitor erythromycin, we found that these non viable cells were more likely to be found in acidic vacuoles than in non acidic vacuoles. This difference was not observed for viable cells, further indicating that the bacteria actively avoid ending up in lysosomal compartments.

*Legionella pneumophila* can survive and multiply both in human macrophages and in *A. castellanii* by inhibiting the fusion of phagosomes with lysosomes, a process that is regulated by the *L. pneumophila dot/icm* genes [39]. However, genes involved in *C. jejuni* invasion and intracellular survival in eukaryotic cells remain poorly studied. Watson and Galan showed that *C. jejuni* internalized into T84 human intestinal epithelial cells resided in membrane bound vacuoles that did not fuse with lysosomes [17]. Interestingly, they found a small proportion of *C. jejuni* residing inside dextran filled vacuoles indicating that not all viable bacteria escape the degradative pathway.

In conclusion, our results show that the massive bacterial invasion of *A. polyphaga* observed in co-cultures with *C. jejuni* requires bacterial viability, indicating that this process is bacterially induced. Furthermore, viable *C. jejuni* could escape degradation in *A. polyphaga* by avoiding localization to lysosomal vacuoles. These results provide deeper insights into the process of internalization and intracellular survival of *C. jejuni* in free living amoebae, an interaction that could have important consequences for *Campylobacter* epidemiology. However, further studies on bacterial and amoebal factors involved in this interaction are needed to better understand this process at a molecular level.
